# Composition and regulation of maternal and zygotic transcriptomes reflects species-specific reproductive mode

**DOI:** 10.1186/gb-2010-11-6-r58

**Published:** 2010-06-01

**Authors:** Shai S Shen-Orr, Yitzhak Pilpel, Craig P Hunter

**Affiliations:** 1 Department of Molecular and Cellular Biology, Harvard University, 16 Divinity Ave, Cambridge, MA 02138, USA; 2 Current address: Departments of Pediatrics and Microbiology & Immunology, Stanford University, Stanford, CA 94305, USA; 3 Department of Molecular Genetics, Weizmann Institute of Science, Rehovot, 76100, Israel

## Abstract

**Background:**

Early embryos contain mRNA transcripts expressed from two distinct origins; those expressed from the mother's genome and deposited in the oocyte (maternal) and those expressed from the embryo's genome after fertilization (zygotic). The transition from maternal to zygotic control occurs at different times in different animals according to the extent and form of maternal contributions, which likely reflect evolutionary and ecological forces. Maternally deposited transcripts rely on post-transcriptional regulatory mechanisms for precise spatial and temporal expression in the embryo, whereas zygotic transcripts can use both transcriptional and post-transcriptional regulatory mechanisms. The differences in maternal contributions between animals may be associated with gene regulatory changes detectable by the size and complexity of the associated regulatory regions.

**Results:**

We have used genomic data to identify and compare maternal and/or zygotic expressed genes from six different animals and find evidence for selection acting to shape gene regulatory architecture in thousands of genes. We find that mammalian maternal genes are enriched for complex regulatory regions, suggesting an increase in expression specificity, while egg-laying animals are enriched for maternal genes that lack transcriptional specificity.

**Conclusions:**

We propose that this lack of specificity for maternal expression in egg-laying animals indicates that a large fraction of maternal genes are expressed non-functionally, providing only supplemental nutritional content to the developing embryo. These results provide clear predictive criteria for analysis of additional genomes.

## Background

Early embryos contain mRNA transcripts expressed from two distinct origins; those expressed from the mother's genome and deposited in the oocyte (maternal) and those expressed from the embryo's genome after fertilization (zygotic). Because these transcripts originate from distinct origins they are subject to distinct regulatory constraints. Maternal transcripts rely on post-transcriptional regulatory mechanisms for spatial and temporal control of their embryonic expression, and thus contain all signals that control their stability, localization and relative accessibility to the translational machinery [1-7]. In contrast, zygotically synthesized transcripts may utilize both transcriptional and post-transcriptional regulatory mechanisms to provide precise temporal and spatial expression.

In all animals surveyed to date, at least 30% of protein-coding genes are detected as expressed during the transition from unfertilized oocyte to early embryo [8-13]. These may be divided into three basic groups. First, those that must be expressed exclusively from either a maternal or a zygotic origin, which include maternally expressed genes required to 'jump start' embryogenesis and zygotically expressed patterning genes whose precocious (maternal) expression would disrupt temporal or spatial developmental events [14]. Second, those that must be expressed by both the mother and the embryo - for example, because of low mRNA stability or because of a change in spatial expression in transition between oocyte and embryo [15]. The last group is those genes that can accommodate either maternal or zygotic expression. It is among this latter gene set that evolution can act to maximize the efficiency, or other such measure, of embryogenesis or oogenesis.

A gene's regulatory architecture reflects the extent and complexity of transcriptional and post-transcriptional gene expression. For example, a gene such as sea urchin *endo-16*, which is subject to complex spatial and temporal regulation at a multi-cellular stage of embryogenesis, contains a large complex intergenic regulatory region [16]. In contrast, a gene such as *Drosophila *Oskar, which is transcribed maternally and subject to multiple levels of post-transcriptional regulation, has a large 3' UTR that controls transcript localization, stability, and translation [17]. Finally, many house-keeping genes are ubiquitously expressed and consequently have relatively simple regulatory needs.

At present, accurately and comprehensively assessing the regulatory architecture of the majority of genes is difficult, as the regulation of only a few has been well-characterized [18]. Yet, in organisms with relatively small genomes (up to 150 Mb), genes expressed in many tissues or involved in complex biological processes have longer than average 5' intergenic regions (IGRs) [19,20] and 3' UTRs [21]. Furthermore, the sizes of these regulatory regions correlate positively with the number of known and/or predicted *cis*-regulatory sites [20-22]. Particularly interesting in the context of our study is the observation that the 3' UTRs of maternal genes in *D. melanogaster *are longer than average, suggesting that they are subject to greater post-transcriptional control [5].

In organisms with larger genomes, such as human, housekeeping genes are flanked by small IGRs [23-25] and are associated with low density of conserved non-coding elements. Conversely, genes neighboring large gene-free regions or having large introns have dense regulatory elements and are associated with developmental functions and tissue specificity [25-27]. To first principles, these observations provide a means to assess a gene regulatory architecture, where the extent of regulation is approximated by the length of the regulatory regions, and the type of the region, IGR or UTR, identifies whether the regulation is, respectively, transcriptional or post-transcriptional.

Here, we assess the differing regulatory constraints between maternal and zygotically expressed genes by analyzing the regulatory architecture of individual genes. To do so, we used mRNA time-course expression data to identify maternal and zygotic genes in worm, fly, fish and mouse (*Caenorhabditis elegans*, *Drosophila melanogaster*, *Danio rerio* and *Mus musculus*). For each data set, at least one time point was collected prior to the start of major zygotic transcription, and at least one time point after [4,9,10,15]. In addition, genome-wide mRNA expression data sets from chicken (*Gallus gallus*) eggs and human oocytes allowed identification of maternally expressed genes in those organisms [12,28]. Comparative analysis of maternal and zygotic genes within an animal reveals the effect of yet undescribed selective evolutionary forces acting to modify the gene regulatory architecture of thousands of genes, as a function of germline versus embryonic transcript synthesis. In contrast, cross-species comparisons allow studying this force and understanding the factors that affect it. These show that this selective force affecting gene regulation at the molecular level is in agreement with the alternative strategies for managing maternal versus zygotic energy expenditures at the physiological level, suggesting the maintenance of a delicate balance between different energy resources utilized to 'jump start' embryonic development.

## Results

### Across the animal kingdom, 3' UTRs of maternally expressed genes are not short, reflecting the requirement for post-transcriptional regulation of maternal genes

**Figure 1:**
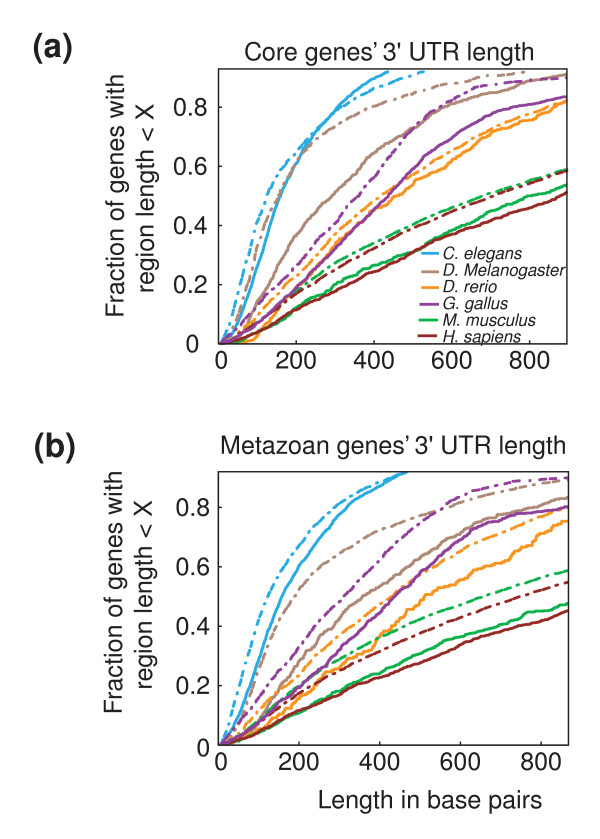
**3' UTRs of maternal genes are under-represented for short lengths**. 3' UTR lengths in six animals comparing all maternally expressed core or metazoan genes (solid curves) versus all other core or metazoan genes in the genome (dotted curves). **(a) **Core genes (minimum *P*-value; percentile at which the minimum *P*-value was detected; top most percentile showing significance): *C. elegans *(*P *< 10^-18^; 20th; 100%); *D. melanogaster *(*P *< 10^-9^, 25th, 100%); *D. rerio *(*P *< 10^-6^, 20th , 85%); *G. gallus *(*P *< 10^-5^, 65th, 100%); *M. musculus *(*P *< 10^-12, ^25th, 100%); *H. sapiens *(*P *< 10^-12^, 25th, 100%). **(b) **Metazoan genes: *C. elegans *(*P *< 10^-26, ^20th, 100%); *D. melanogaster *(*P *< 10^-30^, 35th , 100%); *D. rerio *(*P *< 10^-6^, 45th, 100%); *G. gallus *(*P *< 10^-17^, 40th, 100%); *M. musculus *(*P *< 10^-23^, 20th, 100%); *H. sapiens *(*P *< 10^-18^, 35th, 100%).

Genes whose transcripts were detected as present in the embryo before the initiation of zygotic transcription were defined as members of the 'all-maternal' gene class (see Materials and methods). To compare the relative contribution of post-transcriptional regulation among different classes of maternal transcripts, we used the length of the 3' UTR as an estimate of the complexity of a gene's post-transcriptional program (addition of 5' UTR length yielded qualitatively similar results; see Materials and methods). To account for differences in functional complexity [19-21,26,29], we applied a genome-wide phylogenetic profile of 26 organisms [30] to classify genes as either 'core' (conserved in both uni-cellular and multi-cellular organisms) or 'metazoan', and analyzed them separately. In all animals the 3' UTR lengths of the all-maternal class genes were significantly under-represented for short lengths compared to all other coding genes (Figure 1a, b; *P*-value <0.05 in all cases using a modified Kolmogorov-Smirnov test; see Figure 1 legend and Materials and methods for details). In addition, with the exception of *C. elegans *and *G. gallus*, significant differences were also detected between all-maternal core and metazoan genes. This preservation of 3' UTR length among maternal transcripts occurs across a 30-fold range in genome size (100 Mb to 3 Gb), a 5-fold range in genome-wide mean 3' UTR length (150 to 900 bp), and large differences in development and stability of maternal transcripts [7,31,32]. We conclude that across the animal kingdom the post-transcriptional regulatory constraint imposed on maternally expressed genes has selected against short 3' UTRs.

### *D. melanogaster *zygotic genes have longer 5' IGRs whereas maternal genes are under-represented for short 3' UTRs

After the initiation of zygotic transcription, the assignment of relative maternal and zygotic transcription to a gene's measured mRNA abundance becomes less certain. However, for *D. melanogaster*, exact quantification of relative maternal and zygotic contributions to mRNA abundance was made possible through the use of embryos lacking entire chromosomes [15]. This analysis defined five separate gene classes for transcripts detected in early embryos (see Materials and methods): strict-maternal and strict-zygotic genes are expressed solely from one origin of expression; mostly-maternal and mostly-zygotic genes are those whose expression profile is similar to their strict counterparts, but for whom at least some contribution (less than 33%) is due to zygotic or maternal origin, respectively [15]; and finally, the maternal-zygotic genes are those that are transcribed maternally, but whose transcript abundance level does not change significantly throughout the duration of the experiment (either stable or supplemented by zygotic transcription).

Comparison of 3' UTR lengths between the five different origin-of-synthesis classes showcases the effect of the biological constraints on 3' UTR length. The 3' UTRs of maternal and zygotic class genes are significantly longer than those of other genes in the genome. In particular, with the exception of the core strict-zygotic class, both core and metazoan strict-maternal genes are underrepresented for short 3' UTRs compared to all other classes (Figure S1 in Additional file 1; across all comparisons *P*-value at least ≤ 0.02). Interestingly, the longest 3' UTRs are those of zygotic genes.

**Figure 2:**
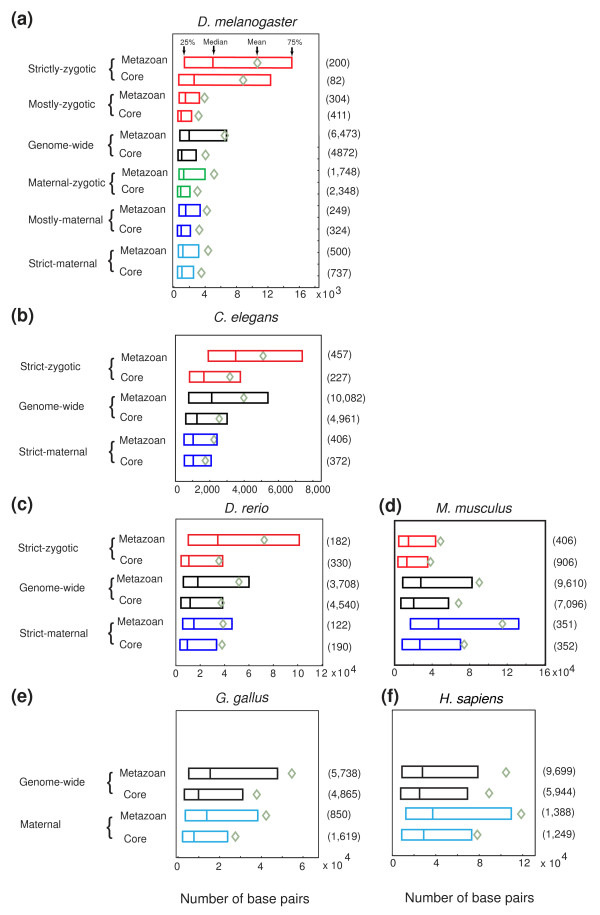
**5' IGR length in all animals is dependent on both gene functional complexity and transcript origin of synthesis**. **(a) **Genetic manipulation of *D. melanogaster *enables quantification of the maternal and zygotic components of mRNA abundance, allowing analysis of five gene classes. Genes expressed solely by the zygote have long 5' IGRs, whereas genes expressed by the mother have short 5' IGRs. Observed differences are greatest when comparing genes expressed exclusively from one origin. **(b-d) **Similar comparisons for *C. elegans*, *D. rerio *and *M. musculus*, where gene classification is based solely on characteristic strict-maternal and strict-zygotic expression profiles. In mouse an inverse relationship between maternal and zygotic genes is observed. **(e,f) **5' IGR length comparison of all maternally expressed genes in *G. gallus *and *H. sapiens* to all other genes in the genome. Like mouse, human maternal genes have large 5' IGRs. In all plots, genes were partitioned to core and metazoan classes by phylogenetic filtering. Core genes have shorter 5' IGRs than metazoan ones. Numbers in parentheses to the right of each box plot bar are numbers of genes per class.

Significant differences are also observed between maternal and zygotic genes with respect to 5' IGR lengths (addition of intron lengths and/or 3' IGR lengths yielded qualitatively similar results; see Material and Methods). For metazoan genes, the four gene classes that include some maternally contributed transcripts have significantly shorter 5' IGR lengths than all other metazoan genes in the genome (Figure 2a; *P *< 10^-9^, *P *< 10^-4^, *P *< 10^-12^, *P *< 10^-5 ^for strict-maternal, mostly-maternal, maternal-zygotic and mostly-zygotic, respectively). Strikingly, the 5' IGR lengths of the small set of 282 genes belonging to the strict-zygotic class are extremely long compared to all other gene sets (*P*-values for core and metazoan genes, respectively, were: strict-maternal, *P *< 10^-5 ^and *P *< 10^-18^; mostly-maternal, *P *< 10^-6 ^and *P *< 10^-12^; maternal-zygotic, *P *< 10^-7 ^and *P *< 10^-18^; mostly-zygotic, *P *< 10^-6 ^and *P *< 10^-13^; the genome-wide set of all core and metazoan genes, *P *< 10^-11 ^and *P *< 10^-10^). Interestingly, this class is enriched for patterning genes (*P *< 10^-32^), whereas the strict-maternal class is enriched for core genes (*P *< 10^-115^) [15], as would be expected from the proposed theory on maternal and zygotic gene expression in rapidly developing organisms [14]. Lastly, comparing the core genes to metazoan genes the 3' UTRs and 5' IGRs of core genes are shorter for nearly all maternal and zygotic classes (*P*-values for 3' UTRs and 5' IGRs, respectively, were: strict-maternal, *P *< 10^-6 ^and *P *< 0.07; mostly-maternal, *P *< 10^-9 ^and *P *< 10^-6^; maternal-zygotic, *P *< 10^-35 ^and *P *< 10^-21^; mostly-zygotic, *P *< 10^-12 ^and *P *< 10^-7^; strict-zygotic, *P *< 10^-4 ^and *P *< 10^-3^; the genome-wide set of all core and metazoan genes, *P *< 10^-21 ^and *P *< 10^-72^).

### Similarity in regulatory architecture of maternal and zygotic genes across the animal kingdom highlights the complexity of regulation of mammalian maternal genes

To analyze the gene architecture of maternal and zygotically expressed genes in other animals (*C. elegans*, *D. rerio*, *G. gallus*, *M. musculus *and *Homo sapiens*) we defined three gene classes for transcripts detected in early embryos: maternal, zygotic and maternal-zygotic. For chicken and human, to the best of our knowledge, only pre-zygotic transcript data are publicly available; thus, for these species we contrasted the all-maternal gene class with the genome-wide set of core and metazoan genes. Further, due to the lack of genetic controls available in *Drosophila*, for these other species we must rely on the characteristic expression profile to define the origin of expression (see Materials and methods). For clarity, we use the nomenclature applied to the *Drosophila *data and refer to the maternal and zygotic gene classes as strict-maternal and strict-zygotic. By necessity, the maternal-zygotic class is less precisely defined and includes slowly decaying strict-maternal genes. Consistent with this, we find that the lengths of the regulatory regions in the maternal-zygotic class are, by and large, intermediate to those observed in the strict-maternal and strict-zygotic gene classes (data not shown). Therefore, unless otherwise noted, we exclude the maternal-zygotic class from further analysis.

Next, for each species we compared the 5' IGR lengths as proxies for the functional complexity of maternal and zygotic gene regulatory regions. Additionally, within these origin-of-synthesis class gene sets, we compared the core and metazoan subclasses to the genome-wide core or metazoan gene sets (see Materials and methods). Because it is meaningless to compare the absolute lengths of genes' regulatory region size across species with vastly different genome sizes, the genome-wide core or metazoan gene sets provide a means to normalize length for cross-species comparisons. Performing this comparative analysis between maternal and zygotic gene classes separates the studied animals into two distinct groups. *C. elegans*, *D. rerio *and *G. gallus *genes show a pattern similar to that described for *D. melanogaster*. The 5' IGRs of *C. elegans *and *D. rerio *strict-maternal genes (Figure 2b,c) are shorter than those of the respective zygotic genes (*P*-values for core and metazoan genes, respectively, were: *C. elegans*, *P *< 10^-10 ^and *P *< 10^-27^; *D. rerio*, *P *< 0.1 and *P *< 10^-3^) while the genome-wide average is intermediate. Similarly, *G. gallus *all-maternal genes' 5' IGRs are smaller than the genome-wide average (Figure 2e; core, *P *< 10^-5^; metazoan, *P *< 10^-3^). Furthermore, *C. elegans *and *D. rerio *maternal and all-maternal gene classes are enriched in core genes compared to the zygotic class (*P *< 10^-147^). This pattern is strikingly reversed in the mammals (Figure 2d, f). Mouse strict-maternal gene 5' IGRs are longer than the genome-wide average (core, *P *< 10^-3^; metazoan, *P *< 10^-7^) while the 5' IGRs of strict-zygotic genes are smaller (core, *P *< 10^-9^; metazoan, *P *< 0.01). Similarly, human all-maternal gene 5' IGR lengths are larger than the genome-wide average (Figure 2f; core, *P *< 0.03; metazoan, *P *< 10^-7^). Unlike the other animals, mouse strict-maternal and all-maternal classes are enriched for metazoan genes (*P *< 10^-226^).

These differences among maternal genes between mammals and the other animals is highlighted by the otherwise consistent relationship observed in all animals of shorter regulatory region lengths for core genes than for metazoan genes (*C. elegans*, *P *< 10^-49^; *D. rerio*, *P *< 10^-17^; *G. gallus*, *P *< 10^-29^; *M. musculus*, *P *< 10^-20^; *H. sapiens*, *P *< 10^-5^). Specifically, as observed in *Drosophila*, the 3' UTRs of core genes are shorter than the 3' UTRs of metazoan genes and the 3' UTRs of strict-maternal and all-maternal transcripts are underrepresented for short lengths (Figure S2 in Additional file 1; Figure 1 for *G. gallus *and *H. sapiens*). Thus, the only significant difference in gene architecture between mammals and the other animals examined here is in the length of the 5' IGRs of maternal and zygotic genes. The relatively large size of mammalian maternal 5' IGRs compared to the genome-wide set suggests that maternal genes in mammals have complex and highly specific transcriptional regulation, whereas maternal genes in the other animals, which are much shorter than the genome-wide set, are regulated with less specificity.

### Mammalian maternal genes are under selective pressure to maintain large 5' IGRs

These observations may reflect either an actual biological difference or a limitation in our definition of maternal and zygotic genes. In all animals, the data for identification of zygotically transcribed genes spanned a time course extending many cell divisions after the start of zygotic transcription, at least up to the metazoan hallmark of gastrulation [4,9,15,33]. It has been suggested that gastrulation, and not fertilization, is the time point best suited for alignment of eutherian development with other metazoans [34]. If true, we would expect mouse zygotic genes that are expressed at or after gastrulation to exhibit increased transcriptional complexity. Interestingly, the density of conserved sequences is high in non-coding regions flanking genes expressed in mouse embryos at 9.5 to 10.5 days of gestation but not earlier in development [25]. Furthermore, genes flanked by gene deserts are enriched in developmental functions in mouse, as well as in human and chicken [26]. This suggests that analysis of IGRs of genes expressed later in mouse development may identify a developmental time point in which the 5' IGRs of the genes expressed will be as long, if not longer, than those of the strict-maternal set. For maternal genes, sparse mRNA abundance measurements may hamper our ability to distinguish strict-maternal-only genes from maternal-zygotic genes.

To confirm that our observations were due to a true biological difference, we compared the all-maternal class from each animal to its respective genome-wide average. For mouse, 5' IGRs of the all-maternal class were larger than the genome-wide average, whereas for all other animals the 5' IGRs of all-maternal genes were statistically significantly shorter than the genome-wide average (Figure S3 in Additional file 1). These observations highlight that the differences observed in the architecture of maternal genes' 5' IGRs, both when compared to zygotic genes within the same animal and when compared across animals, are due to true biological variation.

**Figure 3:**
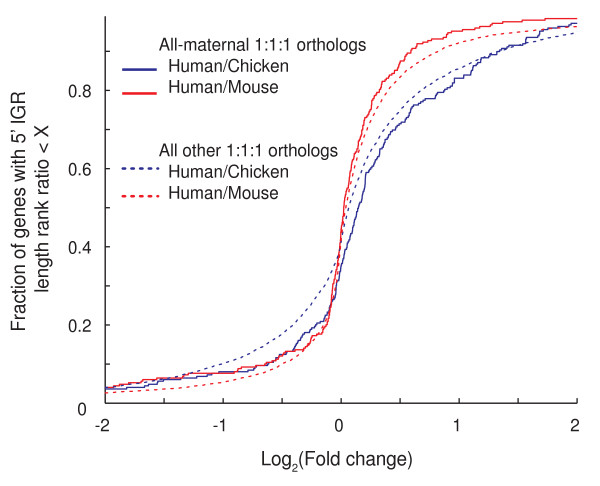
**Systematic change in relative size of 5' IGRs of maternally expressed human and chicken one-to-one orthologs**. Shown is the cumulative distribution of fold-change difference in relative 5' IGR size for all human, chicken and mouse 1:1:1 orthologs (dotted curves) versus those expressed maternally in all three organisms (solid curves). Fold change is shown on a log_2 _axis. A fold change of zero implies that the length of the 5' IGRs of a gene and its 1:1 ortholog ranked the same within their respective genome. Similarly, a positive fold change implies a gene's 5' IGR has expanded in relative size in human (and/or shrunk in mouse or chicken) with respect to the relative size of its ortholog's 5' IGR in mouse or chicken. The converse is implied by negative log_2_(fold change).

The observed differences in gene architecture between mammalian maternal genes and other animals may be due to either the expression of different genes or differing regulatory needs of the same genes. Comparative analysis of relative changes in IGRs of maternally expressed versus non-maternally expressed orthologous genes offers an opportunity to discern the cause of the observed differences. From the animals studied here, *G. gallus *is phylogenetically closest to mammals but, unlike them, its maternal genes have short 5' IGRs. To account for differences in absolute genome size, we normalized and ranked regulatory region lengths and then calculated the ratio of ranks between individual one-to-one ortholog pairs of chicken-human and mouse-human (see Materials and methods). For each orthologous pair we obtained one value representing its fold change in percentile ranking of IGR length between chicken and human, and another for its fold change between mouse and human. Comparison of fold changes of all-maternal one-to-one orthologs versus the set of all one-to-one orthologs shows a shift towards larger fold changes in human to chicken (Figure 3, blue lines; *P *< 0.01). However, calculating this ratio for mouse versus human genes showed no statistically significant fold changes (Figure 3, red lines). This implies that the 5' IGRs of maternally expressed genes in human and mouse have expanded more than would be expected given the genome sizes or that chicken maternally expressed genes have shrunk. Coupled to the observation that oocyte deposited transcripts in chordates are highly conserved [35], we conclude that the difference in maternal genes' 5' IGR lengths between mammals and other animals may be due to selection for complex transcriptional regulation of mammalian maternal genes.

## Discussion

The variations observed across six animals in 5' IGR and 3' UTR lengths provide an opportunity to understand the evolutionary pressures shaping maternal and zygotic genes. To do so, we have relied on the amassed knowledge that precise gene regulation in space, time and abundance requires complex regulatory regions [36], which, in turn, require more genomic real estate [19,20,37,38]. Our observations that in every animal studied here, the regulatory regions of maternal or zygotic core genes are shorter than those of the respective metazoan genes support this notion.

*D. melanogaster *maternal genes have previously been reported to have significantly longer 3' UTRs than non-maternal genes [5]. However, our meta-analysis of early embryogenesis in six different species suggests that this statement is inaccurate in a subtle but important manner. Specifically, our analysis suggests that the universal pattern for 3' UTRs of maternal genes is that they are not longer than zygotic genes, but rather for both core and metazoan classes are underrepresented for short lengths. This suggests that the post-transcriptional regulatory constraint imposed on maternally expressed genes has functioned to maintain 3' UTR lengths across the animal kingdom [1-3,6,7]. For maternal genes, transcriptional regulatory mechanisms cannot specify spatiotemporal expression patterns; therefore, any maternal gene that shows complex expression must employ a post-transcriptional regulatory program. Conversely, this regulatory constraint on 3' UTRs of maternal genes does not convey any knowledge of the complexity of the regulatory program or require that zygotic genes not utilize post-transcriptional regulatory mechanisms. This is best observed in the De Renzis *et al*. [15]*D. melanogaster *data set, in which the maternal and zygotic contributions are precisely determined by genetic decoupling (Figure S1 in Additional file 1). However, it is also apparent in our analysis of *C. elegans *(Figure S2a,b in Additional file 1) and *D. rerio *metazoan genes (Figure S2b in Additional file 1), in all of which the longest 3' UTRs belong to strict-zygotic metazoan genes, in agreement with recent work on the role of microRNAs in embryonic development [21,22,39].

In contrast, analysis of maternal and zygotic gene 5' IGRs yielded a dichotomy between mammals and the other animals. Given the highly conserved relationship between core and metazoan genes with regard to 5' IGR regulatory region size, what explains the divide in transcriptional specificity when it comes to transcriptional regulation of maternal genes? An appealing possibility is that differences in gene architecture are mirroring differences in development, specifically pre- and post-fertilization dynamics. We note that the divide in relative 5' IGR size precisely matches the species mode of reproduction. Those with relatively short 5' IGRs are all egg laying, oviparous animals, whereas those with relatively long 5' IGR length are the viviparous mammals. An important difference between oviparous and viviparous animals that is likely to affect gene architecture is the temporal constraint on maternal contributions to the embryo, which for oviparous species ceases at fertilization, while in the viviparous species continues post-fertilization. To our knowledge, the only other developmental characteristic that corresponds to the differences in regulatory region size is that many oviparous embryos begin development with a series of rapid cellular cleavages, while in mammals the initial cell cycles are slow, with rapid cleavages occurring only later [34]. Indeed, in animals where initial cleavage divisions are rapid, early zygotic genes often have small or no introns [15], a gene architectural feature important for producing a functional transcript during these abbreviated cell cycles [40]. However, the 5' IGR is not transcribed and transcription of the maternal genes occurs before these rapid cleavages; thus, the rapid early development can have only an indirect effect on maternal gene architecture.

**Figure 4:**
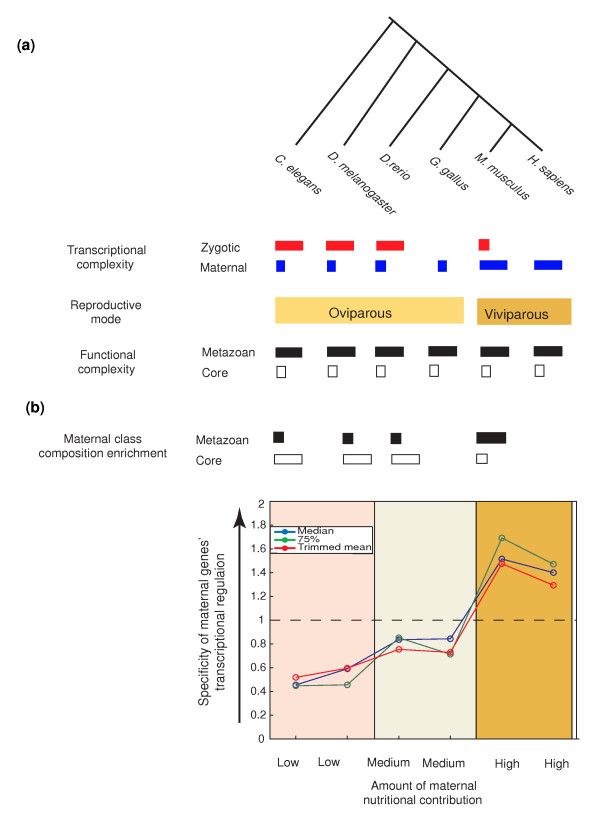
**Specificity of expression of maternally expressed genes correlates positively with the amount of maternal nutritional contribution**. **(a) **Schematic summarizing the size of transcriptional regulatory regions of maternal and zygotic genes in each species, relative to one another and to the genome-wide average. We note a dichotomy that matches the reproductive mode. The highly conserved relationship between core and metazoan genes' relative 5' IGR regulatory region size suggests that regulatory region length may be considered a metric for complexity and specificity of transcriptional regulation. **(b) **Organizing animals by the amount of nutritional contribution provided by the mother (low, medium, high), we estimate the specificity of maternal gene expression by the ratio of maternal metazoan gene 5' IGR length to the genome average. Shown are three measures of the ratio of maternal to genome-wide regulatory region lengths for strict-maternal genes (for *G. gallus *and *H. sapiens *all maternally expressed genes). Comparison is restricted to metazoan genes, as they comprise the subset most reflective of changes in regulatory complexity.

One mechanism by which developmental constraints, such as rapid early development or a prolonged pre-fertilization stasis, can affect gene architecture is by the selection for or against expression of specific gene classes in either the oocyte or embryo. Wieschaus [14] has proposed that gene expression is a limiting resource in rapidly developing oviparous animals. Under this hypothesis, those genes whose expression can be accommodated from either maternal or zygotic origin will, over evolutionary timescales, shift to maternal expression. This will relieve the embryo from the synthetic cost (energy and time) to express those genes, thereby minimizing the time to hatching and maximizing the competitive advantage for limited environmental resources. In the extreme, the only transcripts to be expressed zygotically would be those providing spatial and temporal patterning information or whose precocious expression would disrupt early events [14]. The analysis of the high resolution *D. melanogaster *data set is fully consistent with this hypothesis. Strictly zygotic genes are highly enriched for patterning genes. Similarly, we detect a strong enrichment for metazoan functions, including patterning, in the other oviparous species we analyzed. Furthermore, *D. melanogaster *strictly zygotic genes have very large regulatory regions, much larger than the genomic average or even of other developmental genes (strict-zygotic versus developmental genes: core, *P *< 0.09; metazoan, *P *< 10^-4^; data not shown). The insight we gain into complex regulation and specificity from the analysis of core and metazoan genes suggests that the expression of these strictly zygotic genes is temporally and spatially complex. On the other hand, the 5' IGR length (but not 3' UTRs) of maternally expressed genes (including maternal-zygotic and mostly-zygotic) is dramatically shorter than the genomic average, suggesting reduced regulatory specificity. Again, we observe the same phenomena in the other oviparous species for which zygotic gene data are available (Figure 4a).

Wieschaus hypothesized an efficiency-based shift towards maternal gene expression for fast-developing oviparous organisms [14]. However, based on our data we propose that the shift, under certain conditions, can be towards zygotic gene expression. Specifically, viviparous animals develop relatively slowly and the embryo competes for limited environmental resources only via the mother. In contrast, the relatively undifferentiated mammalian oocyte needs to persist indefinitely, and thus may be under selective pressure to minimize energy expenditures and thus maximize gene expression specificity (larger 5' IGRs). Thus, selection for efficiency may generate complex 5' IGRs relative to the genome-wide average for viviparous maternal genes and for oviparous zygotic genes.

One of the most striking features of our analysis is the low complexity of 5' IGRs of maternal genes relative to the genome-wide average in oviparous animals. This feature is only partially explained by a shift in functional composition, as it occurs for both core and metazoan gene subclasses as well as in one-to-one orthologs (Figure 3). We consider two hypotheses to explain this. The first is tolerated profligate expression. The apparently low threshold for maternal expression may enable many genes, over evolutionary time, to non-functionally sample maternal expression. Over time, maternal expression of developmentally neutral genes will accumulate. However, this hypothesis does not explain the apparent selection for non-short 3' UTRs, which suggests selection for post-transcriptional regulatory information. Thus, we propose a second hypothesis: maternal contributions to embryonic development also include energy and nutrition. Mammals rely on lactation and placentation, while oviparous animals deposit yolk, consisting mainly of proteins, lipids, and phosphorous, into oocytes. The non-functional maternal transcripts provide nutrient stores of nucleotides and phosphate for the rapidly developing embryos. Our data show a positive correlation between maternally provided nutrition (low for worm and fly, higher in zebrafish and chicken, and highest in mammals) and the complexity of maternal gene regulation (Figure 4b). Since maternal transcripts also provide a low osmotic store of nucleotides and phosphate, they may be considered nutritional. Thus, it is possible that some maternal transcripts are purely nutritional. Such a hypothesis suggests that 'misexpressing' a gene in the maternal germline should not be associated with an energy or efficiency cost. Rather, such 'profligate' expression of non-detrimental transcripts may be advantageous and selected for. Furthermore, such a selective force could provide a mechanism for creation of new non-coding RNA genes that could evolve into coding genes or exons.

These two interpretations, developmental constraints and nutrient stores, present three testable predictions. First, both models predict a bias in gene function between genes expressed maternally and genes expressed zygotically. For example, consider a gene that is not selected for either a maternal or a zygotic mode of expression. The expectation is that expression of that gene will drift between strict maternal and strict zygotic expression, such that, at any given time, a set of such genes would be equally represented in both groups. Thus, any bias in the distribution indicates non-neutral evolution, either by functional restriction or gene flow based on energy and timing considerations as described above. Indeed, as we noted above, we observed maternal depletion/zygotic enrichment of metazoan-specific genes, which are enriched for patterning functions, in fast developing embryos (Figure 4b).

Second, the nutrient stores model predicts enrichment for expression of non-functional maternal genes in organisms with limited maternal nutritional contributions (yolk). This is based on the positive correlation we observe between the amount of yolk and the simplicity of maternal gene expression, suggesting that maternal gene regulation becomes promiscuous as maternal nutritional contributions are limited (Figure 4b). Consistent with this, many maternally expressed *C. elegans *and *D. melanogaster *genes do not have an apparent phenotype by RNA interference knockdown [41-43]. In support of this, we tested for regulatory region length differences between *C. elegans *maternal genes for which an RNA interference (RNAi) phenotype is detected and those for which it is not (see Materials and methods). Significant differences were detected in 5' IGR lengths (*P *< 10^-8^), but not 3' UTRs (Figure S4 in Additional file 1).

Third, we predict that the constituency and regulation of maternal and early zygotic transcripts will only mirror phylogeny to the extent that it agrees with forms of maternal contribution. Viviparity and oviparity have developed multiple independent times, in various forms, in distant branches such as arthropods, sharks, lizards and eutherian mammals. Based solely on the extent of maternal contribution, our results predict not only how early developmental genes would be regulated in marsupials and monotreme species, relatively close to the studied mammals, but also that the regulation of genes in early development would be more similar between two distant viviparous animals than between closely related animals with differing reproductive modes.

## Conclusions

Here we analyze the regulation constraints of the maternal-zygotic transition, a key developmental process in all animals, involving thousands of genes. The utilization of regulatory region lengths to study complex molecular processes circumvents the present deficiency in detailed information on individual gene regulation and offers a clear methodology for study of other, so-far undecipherable biological processes. Importantly, as a baseline control, we show that differences in the inferred lengths of regulatory regions between different functional gene classes are conserved, irrespective of genome size. At a time when new, non-model organisms' and unannotated genomes are being sequenced at an ever-increasing rate, such methodologies are required to identify and study genes in these organisms.

Our comparative analysis of maternal and zygotic genes within an animal reveals that the location and abundance of regulatory content is driven by at least two forces: one reflected in the inferred functional complexity of gene action [19,21], and a second related to the origin of synthesis of transcripts. This latter selective evolutionary force is acting to modify, as a function of germline versus embryonic transcript synthesis, the gene regulatory architecture of thousands of genes. In contrast, cross-species comparisons allow analyses of this force and suggest that it is coupled to the timing of the maternal-zygotic transition, which correlates with alternative strategies for managing maternal versus zygotic energy expenditures at the physiological level. Taken together, these results uncover an ancient force affecting the development of all multi-cellular organisms and provide clear predictive criteria for the nature of maternal-zygotic gene regulation in other animals.

## Materials and methods

### Classification of genes to maternal and zygotic classes

Gene identifiers, chromosomal locations and sequences for all organisms were mined from EnsEMBL V42 December 2006 [44] and Wormbase (release WS160). To classify genes to either maternal or zygotic origin, we used the expression data sets of Baugh *et al*. [9], De Renzis *et al*. [15], Giraldez *et al*. [4] and Hamatani *et al*. [10] for *C*. *elegans*, *D*. *melanogaster*, *D. rerio *and *M. musculus*, respectively. To identify maternal genes in *H. sapiens *and *G. gallus*, we used the expression data of Kocabas *et al*. [12] and Lee *et al*. [28]. See Additional file 1 for a detailed description of how maternal and zygotic genes were identified from each of these data sets.

For *C. elegans*, maternal and zygotic classes correspond respectively to the strictly maternal degrading and strictly embryonic classes [9]. For *D. melanogaster*, De Renzis *et al*. [15] reported, at a fold change of three and a *P*-value < 0.001, 6,485 maternally expressed genes, of which 2,110 decreased significantly in their abundance during the time course. Of these 2,110 genes, 633 had a significant zygotic component contributing to their measured abundance level (Table S7 in De Renzis *et al*. [15]). We considered the 6,485 genes as all-maternal and the 1,477 maternal decreasing genes with no zygotic component as strict-maternal. For the zygotic class, we used the 334 genes expressed at cycle 14 with no maternal contribution (Table S4 in De Renzis *et al*. [15]). The remapping of genes to FlyBase 4.3 reduced the number of genes in each class to 5,923, 1,358 and 314 for all-maternal, strict-maternal and zygotic, respectively. For *D. rerio *we used the Giraldez *et al*. [4] classification of *D. rerio *genes as 'predominantly maternal' and 'predominantly zygotic' as 'maternal' and 'zygotic' classes, respectively [4]. Briefly, genes expressed at 1.5 hours post-fertilization and showing a significant reduction at 50% and 90% epiboly were considered maternal. Genes expressed significantly at the 50 and 90% epiboly stages and not at 1.5 hours post-fertilization were considered zygotic. For *G. gallus*, we considered the top ranked 50% of expressed genes of stage X embryos (a laid egg) as maternal. In stage X eggs, an undifferentiated blastoderm has formed on top of the yolk, but major zygotic activation has yet to occur [45]. Results did not change if we set the threshold to a more restrictive 25%, but the number of genes was reduced, which affected our orthologous gene comparisons (see below). For *M. musculus *[10], genes mapping to clusters 7 or 9 were considered 'maternal' and genes mapping to clusters 1, 4, 5 and 8 as 'zygotic'. To classify which genes were expressed during gastrulation, we ranked genes detected as expressed in wild-type embryos from 6.5 days post-cleavage [33]. The top 25% expressed genes were considered zygotic. Varying this threshold from 5 to 50% did not change our results. The 5,331 transcripts identified by Kocabas *et al*. [12] as up-regulated in *H. sapiens *MII oocyte transcripts were considered maternal. To the best of our knowledge, a quality data set identifying human zygotic genes is not available. For each organism the genome-wide gene set was defined as all genes in the genome that meet the criteria (as defined in the 'Classification of genes to core and metazoan classes' and 'Estimates of regulatory region lengths' sections below) to be included in the analysis (for example, no downstream operon genes were included in the *C. elegans *genome-wide set when calculating the distribution of genome-wide 5' IGR lengths).

### Classification of genes to core and metazoan classes

We used the Inparanoid: Eukaryotic Ortholog Groups database (release 5.1, January 2007) [30] to classify genes into core and metazoan classes by phylogenetic profiling. This version of Inparanoid contains an all-against-all protein coding gene blast comparison of 26 organisms - 1 prokaryote, 3 unicellular eukaryotes, 2 plants and 20 metazoans (including a urochordate, nematodes, insects, fish, bird, amphibian and mammals) [30]. A core gene was defined as any gene present in one or more of the unicellular organisms included in InParanoid. A metazoan gene was defined as any gene present in two or more animals included in Inparanoid that is not present in the core gene set or in plants. The organisms used to define the core gene set are *Escherichia coli*, *Saccharomyces cerevisiae*, *Schizosaccharomyces pombe *and *Dictyostelium discoideum*.

We tried several different criteria (higher and lower) for the metazoan gene set definition, and obtained similar qualitative results with different values of significance. For *C. elegans *and *D. melanogaster *we repeated our analysis using the classification scheme defined by Nelson *et al*. [19], which classifies genes by their expected regulation complexity (simple or complex) based on their molecular functions and the biological processes they are involved in. For *C. elegans *we updated the gene annotations directly from Wormbase GO (release WS150). For both species, all results obtained from this analysis were qualitatively the same as those obtained from the phylogenetic profiling data set.

### Estimates of regulatory region lengths

We defined a gene's 5' IGR length as the distance between its 5'-most coding nucleotide and the closest respective upstream or downstream coding nucleotide belonging to a different gene on either DNA strand. Similarly, 3' IGR length was calculated as the distance from the 3'-most stop codon to the downstream closet coding nucleotide belonging to a different gene. We defined the first intron as the intron closest downstream to the translation start site. To estimate first intron lengths, we used two measures: the length of the largest first intron of a gene among all the first intron lengths of its alternative splicings, and the largest continuous non-coding segment in the first intron. Both intron length measurement types yielded similar results. In *C. elegans*, for genes transcribed as a part of an operon, only the 5'-most gene (first gene) was included in any analysis involving 5' IGR length.

The length of a gene's 3' UTR was approximated as the maximum 3' UTR length of all of its alternatively spliced transcripts. A similar calculation was performed for 5' UTRs. We considered the sum of both 3' and 5' UTRs as the total post-transcriptional regulatory region size for all animals except for *C. elegans*, where post-splicing makes this metric moot. A large fraction of genes in any given genome are annotated with either no UTR information or with a UTR that is only a few base pairs long. We noticed that this UTR annotation is replaced with full length UTRs with successive updates of the database and hence appears to be missing or have incomplete annotation. No significant enrichment in extremely short UTRs (less than 5 bp) was detected for either core, metazoan, maternal or zygotic genes; however, their inclusion in the analysis shifted the mean/median of the distributions greatly due to their large numbers. Thus, we placed a lower bound on UTR length, considering them as artifacts, and discarded any 3' UTRs shorter than 5 bp and any 5' UTRs shorter than 3 bp in all species.

We calculated 3' UTR lengths twice, once allowing for multiple exons in the 3' UTR and once without. Roughly 10% of reported 3' UTRs in every organism have multiple 3' UTR exons, which are thought to be subject to non-sense mediated decay degradation [46] - statistical tests and plots appearing here are all for 3' UTRs, which do not contain multiple exons - but results are qualitatively the same when allowing for multiple exons in 3' UTRs. For zebrafish, only genes having a RefseqId [47] were included in the analysis of 3' UTR lengths.

To determine that our results are robust to exact definitions of regulatory region lengths, we considered for both transcriptional and post-transcriptional regions alternative definitions of a genes' regulatory regions. For transcriptional regulatory region length comparisons between gene groups, we performed our analysis using not only 5' IGRs, but also the total length of a gene's 5' IGR plus the first intron, the sum of IGRs (5' IGR plus 3' IGR), and the sum of all three (5' IGR plus first intron plus 3' IGR). For post-transcriptional regulation we estimated the 3' UTR length as well as the total sum of UTRs (5' plus 3'). Transcriptional regulatory region estimates across all genes showed that they were highly correlated with one another (Figure S5a in Additional file 1). Similarly, the two post-transcriptional regulatory region estimates were also highly correlated with one another (Figure S5b in Additional file 1). We applied the analyses presented here using each of the different estimates of transcriptional and post-transcriptional regulatory region length for each of the species. Analysis of each of these for every species yielded qualitatively the same results. The 5' IGR plus the first intron analysis mirrored very closely the observed signal in the 5' IGR, whereas analysis of regions that included the 3' IGR showed reduced, but still significant, differences between regions. Similarly, considering the sum of the 5' UTR and 3' UTR regions for post-transcriptional regulation yielded similar results qualitatively and significance wise. Thus, the results of the analyses we present are robust to the exact definition of regulatory region length, at least to a degree matching the present knowledge of the location of a gene's regulatory information.

### Differences in regulatory region lengths between gene classes

Differences in distributions of the different maternal and zygotic classes were quantified using the non-parametric, one-sided two-sample Kolmogorov-Smirnov test at a significance level α = 0.05. The Kolmogorov-Smirnov test tests the null hypothesis that two sample distributions are drawn from the same distribution and does so by quantifying the distance between the two empirical cumulative distributions. For a given comparison of two distributions, the reported significant *P*-values for this one-sided test indicate that the first distribution of regulatory region lengths under evaluation is shifted to the right (that is, fewer shorter lengths) of the second. For both transcriptional and post-transcriptional regulatory regions, we performed this test once when considering all (100%) regulatory region lengths within each group.

In addition, to quantify the extent that maternal UTRs are under-represented for short lengths, we iteratively applied a one-sided two-sample Kolmogorov-Smirnov test on defined subsets of the distributions. The subsets were determined empirically, beginning with the 15th percentile and incrementing by 5%. For each comparison we report the top most percentile that produced a *P*-value <0.05 and we identify the percentile at which the minimum (most significant) *P*-value was detected. In the text we report only the top-most significant percentile, whereas in the Figure 1 legend we report the most significant *P*-value and the accompanying percentile as well as the top-most significant percentile.

### Orthologous gene analysis between chicken and mammals

To account for differences in genome size and gene number within one genome, we rank-ordered and normalized all genes by 5' IGR length. We then identified all genes with single orthologs in human, mouse and chicken (1:1:1) using Inparanoid. For these, we calculated the ratio of 5' IGR length ranks between every human gene and its one-to-one orthologous chicken counterpart. This ratio represents the fold change in percentile ranking. This procedure was repeated for human and mouse genes. For both chicken-human and mouse-human, these were then divided into those orthologs classified as all-maternal in both species and the remaining orthologous genes. Thus, for every gene we obtained one value representing its fold change in percentile ranking between chicken and human, and another for its fold change between mouse and human.

### Developmental constraints and nutritional/promiscuity model analysis

To perform this analysis, animals were placed into one of three classes (low, medium, high), based on the estimated nutritional contribution provided by the mother. This was estimated from the ratio of the size of an oocyte to the size of an embryo at gastrulation. For each animal the extent of maternal gene transcriptional regulatory complexity is estimated by the ratio of maternal metazoan gene 5' IGR length to the genome average. We restricted our comparison to metazoan genes as they comprise the subset most reflective of changes in regulatory complexity. To calculate the ratio of maternal to genome-wide regulatory region lengths for strict-maternal genes, we used three different measures, including the median of each gene class, the 75th percentile and a 5% trimmed mean. For *G. gallus *and *H. sapiens *we used the all-maternally expressed genes as a substitute for a strict-maternal class, which has not been defined.

To test for differences in regulatory region length between *C. elegans *maternal genes for which an RNAi phenotype is detected and those for which it is not, we obtained from Wormbase WS200 a list of 700,000 *C. elegans *RNAi tests of function, each of which was annotated as to whether a phenotype was observed or not. Cross-checking this against the maternal gene expression list yielded 114,789 that were performed on one of the 5,591 all-maternally expressed genes. Classifying these genes by whether or not a phenotype was observed for them in an RNAi experiment yielded 922 genes that showed no observed phenotype (presumed non-functional maternal genes) and 4,669 with one or more functional (with phenotype) maternally expressed genes. Regulatory region length comparisons between the two groups were performed as detailed in the 'Differences in regulatory region lengths between gene classes' subsection of Materials and methods.

## Abbreviations

Bp: base pair; IGR: intergenic region; RNAi: RNA interference; UTR: untranslated region.

## Authors' contributions

SSO conceived, designed and performed the study, analyzed the results and drafted the manuscript. YP participated in the study design, analysis of the results and drafting of the manuscript. CPH conceived and designed the study, analyzed the results and drafted the manuscript. All authors read and approved the final manuscript.

## Supplementary Material

Additional file 1 **Additional documentation and figures**. Click here for file
