# Supplementary material for: Composition and regulation of maternal and zygotic transcriptomes reflects species-specific reproductive mode
Source: Genome Biol. 2010 Jun 1;11(6):R58. doi: 10.1186/gb-2010-11-6-r58 (PMC2911106; doi:10.1186/gb-2010-11-6-r58)
Supplement: Additional file 1 — Additional documentation and figures. [file gb-2010-11-6-r58-S1.PDF]

Additional Documentation: Composition and Regulation of Maternal and Zygotic Transcriptomes Reflects Species Specific Reproductive Mode

Shai S. Shen-Orr[1†], Yitzhak Pilpel[2], Craig P. Hunter[1§]

[1] Department of Molecular and Cellular Biology, Harvard University, 16 Divinity Ave., Cambridge, MA, 02138, U.S.A

[2]Department of Molecular Genetics, Weizmann Institute of Science, Rehovot, 76100, Israel

[†] Present Address: Departments of Pediatrics and Microbiology & Immunology, Stanford University, Stanford, CA 94305, USA

[§]Corresponding author

This additional documentation contains:

1) Additional figures and legends
2) Detailed description of the choice of public datasets and genes used for the analysis described in the main text.

Additional figure legends

Figure S1 - Comparison of 3' UTR lengths for five maternal and zygotic classes of *D. melanogaster* genes. The 3' UTRs of maternal and zygotic class genes are significantly longer than those of other genes in the genome. Both core and metazoan strict-maternal genes are underrepresented for short 3' UTRs compared to all other classes (Strict-maternal versus Mostly-maternal [Core: $p<0.02$, Metazoan: $p<10^{-6}$]; maternal-zygotic [$p<10^{-24}$, $p<10^{-8}$]; mostly-zygotic [$p<10^{-24}$, $p<10^{-8}$]; strictly zygotic [Not Signifcant, $p<10^{-5}$]; and all coding genes [$p<10^{-30}$, $p<10^{-43}$]), yet the longest 3' UTRs are those of zygotic genes. (a) Core and (b) Metazoan genes.

Figure S2 - Comparison of maternal vs. zygotic 3' UTR lengths for *C. elegans*, *D. rerio*, and *M. musculus* genes. 3'UTRs of maternal genes are underrepresented for short lengths; however, in all animals but mouse, the longest 3' UTRs are zygotic. (a) Core genes in *C. elegans:* [Vs. Genome-wide $p< 10^{-15}$,30%, 100% ; Vs. Zygotic $p<10^{-14}$,40%, 100%], *D. rerio*: [$p< 10^{-4}$,20%, 70% ; Not significant] and *M. musculus*: [$p< 10^{-12}$,40%, 100% ; $p<10^{-17}$,45%,100%], (b) Metazoan genes in *C. elegans:* [$p< 10^{-9}$,25%, 100% ; Not significant], *D. rerio*: [$p< 10^{-6}$,45%, 100% ;Not significant] and *M. musculus*: [$p< 10^{-19}$,25%, 100% ; $p<10^{-9}$,55%,100%].

Figure S3 - Genes expressed in mammalian oocytes have large 5' IGRs. Comparison of 5' IGR lengths of all-maternal core and metazoan genes to the genome wide average. The 5' IGRs of maternally expressed genes in mouse are larger than the genome average (Core: $p< 0.05$, Metazoan: $p<10^{-4}$). This is the opposite of the relationship observed in *C. elegans, D. melanogaster*, and *D. rerio,* (see Figure 2) where the 5'IGRs are smaller than the genome wide average (*C. elegans* [Core: $p<10^{-15}$, Metazoan: $p<10^{-17}$], *D. melanogaster* [$p<10^{-6}$, $p<10^{-17}$], *D.*

rerio [$p<10^{-3}$, not significant for metazoan genes]). This analysis illustrates that the phenomena of large 5’ IGRs in mammals is independent of the definition zygotic activation of transcription.

Figure S4 – Maternal genes with no RNAi phenotype have significantly smaller 5’ IGR. *C. elegans* all-maternal genes were segregated into two groups based on whether or not a phenotype was observed when they were knocked-down by RNAi. This yielded 922 non-functional (no phenotype) and 4669 functional (with phenotype) maternally expressed genes. In agreement with the nutritional/developmental constraints model, (A) maternal genes with no discernable RNAi phenotype showed significant smaller 5’ IGR lengths ($p<10^{-8}$). Yet, (B) no significant differences in 3’ UTR lengths were detected, suggesting that all maternal genes, irrespective of function, are regulated post-transcriptionally.

Figure S5 - Different regulatory regions length metrics are highly correlated in all animals. All *D. melanogaster* core and metazoan genes are sorted by their (A) 5’ IGR length (top panel). Lengths were normalized to between 0 and 1 by the gene with the longest 5’ IGR. Shown below are normalized alternative regulatory region sizes we considered as a proxy for transcriptional regulation complexity, sorted by 5’ IGR lengths. These include: the sum of the 5’ IGR and 1st intron, the sum of the 5’ IGR and 3’ IGR or the sum of the 5’ and 3’ IGRs as well as the 1st intron. ρ denotes the Spearman’s rank correlation between the 5’ IGR and each of the alternative transcriptional regulatory region size metrics. (B) Same as (A) above only for post-transcriptional regulatory region complexity, for which we considered 3’ UTRs (top panel) and the sum of the 5’ and 3’ UTRs. Similarly high correlations to the ones shown here were observed in all other studied species: *C. elegans*. [5’ IGR + 1st intron ρ=.96, 5’ +3’ IGR ρ=.80, 5’IGR + 1st intron + 3’ IGR ρ=.79; 5’+3’ UTR Not applicable], *D. rerio* [ρ=.96, ρ=.72, ρ=.71; ρ=.95], *G. gallus* [ρ=.96, ρ=.81, ρ=.80; ρ=.92], *H. sapiens* [ρ=.96, ρ=.82, ρ=.81; ρ=.90].

Detailed description of the datasets and genes used for the analysis described in the main text

All EnsEMBL data was mined using the EnsEMBL perl API [44]. Operon classification for *C. elegans* was obtained from Wormbase (Wormbase, Release WS160). For the worm data set [9], we remapped the custom designed Affymetrix probes to Wormbase gene annotation (Wormbase, Release WS160) and considered only probes which map to single genes. If multiple probes exist per gene, we only used the data obtained from the single gene probe mapping to the 3' most part of said gene. Thus of the reported 8890 reproducibly detected probes we used only 8080 probes each with a 1:1 mapping to a gene.

The *D. melanogaster* dataset [15] was obtained using the Affymetrix Drosophila genome I array which covers 13,500 genes based on Flybase version 1 models. Many gene models have changed since that version 1 of Flybase and so we restricted ourselves to analyzing genes which we could map to FBIds of Flybase, Release 4.3 and having a CG model number. We also performed all of the presented analysis using the embryonic portion of the Arbeitman et al. [8] dataset. All results obtained were qualitatively the same but usually with a lower significance due to the small number of genes covered by the array. In this case we defined zygotic genes as all those expressed during embryogenesis but not detected maternally. Our choice to present the analysis using the De Renzis et al. dataset is due to its higher coverage of the genome and the ability to separate zygotic and maternal contributions to transcript abundance level.

The *D. rerio* array used to detect expressed genes covers less than half of all predicted genes [4]. Only genes present in contigs that have been mapped to chromosomes were included. We performed an analysis comparing zebrafish maternal and zygotic genes using a different dataset

published by Mathavan et al.[11]. This yielded qualitatively similar results, with a higher significance of differences between 5’ IGR of maternal and zygotic genes, but a lower significance in 3’ UTRs. Since the results published by Mathavan et al. were obtained using a two-channel cDNA array with pooled RNA from all developmental stages as reference, we elected to use the Giraldez et al. dataset instead.

The preimplantation *Mus musculus* gene expression data [10] was generated on the custom NIA 22K 60-mer Oligo Microarray. We used Entrez gene identifiers to map microarray probes to specific mouse EnsEMBL genes, and did not consider probes for which no mapping was found. Probes mapping to multiple genes were not considered. For genes that mapped to multiple probes, a single probe was selected for maternal/zygotic classification, if the expression profile of all probes mapped to the same mega-clusters (See section on Classification of genes to maternal and zygotic classes in Materials and Methods of the main text). This reduced the probe number to 7187 each of which maps to a single gene covering 30% of all protein coding genes in the mouse genome. However, the NIA 22K 60-mer Oligo microarray is enriched for genes expressed in stem cells and preimplantation embryos [10] so we expect that our results are based on a higher proportion of genes expressed in the oocyte than the 30% of the covered genome. To detect which genes are expressed during mouse gastrulation we used wild type samples from dpc 6.5 of mouse development generated as controls in a study conducted by Morkel et al. [33]. The array used in this work is the Affymetrix murine 11K which has been designed based on Unigene Build 4. Mapping Affymetrix probeset names to NCBI assembly 36 of the mouse genome identifies 6040 genes covering roughly 24% of mouse coding genes. We eliminated probes not matching EnsEMBL V45 genes as well as probes mapping to more than one gene and averaged

probes mapping to the same gene. Finally we calculated the mean expression value for every gene across the three hybridizations.

Kocabas et al.[12] profiled human oocytes of young reproductively healthy females using the Affymetrix U133 Plus 2.0 Genechip, which covers the overwhelming majority of protein coding genes in the human genome and a pool of 10 normal tissue samples as reference. Probesets were mapped to EnsEMBL genes.

Lee et al. [28] profiled Eyal-Giladi and Kochav Stage X embryos (a laid egg) on Affymetrix chicken genome gene arrays containing probes for all chicken coding genes. We eliminated probes not matching EnsEMBL V45 genes as well as probes mapping to more than one gene and averaged probes mapping to the same gene.

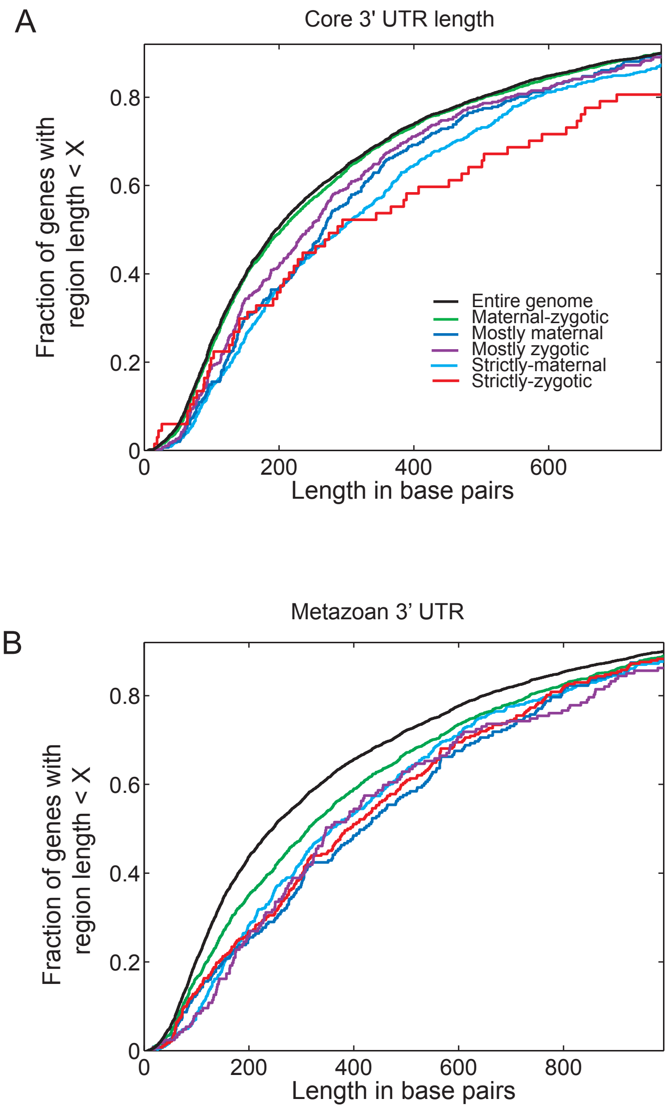
A
Core 3' UTR length
Fraction of genes with region length < X
Length in base pairs
Entire genome
Maternal-zygotic
Mostly maternal
Mostly zygotic
Strictly-maternal
Strictly-zygotic
0
0.2
0.4
0.6
0.8
0
200
400
600
B
Metazoan 3' UTR
Fraction of genes with region length < X
Length in base pairs
0
0.2
0.4
0.6
0.8
0
200
400
600
800

Figure S1.

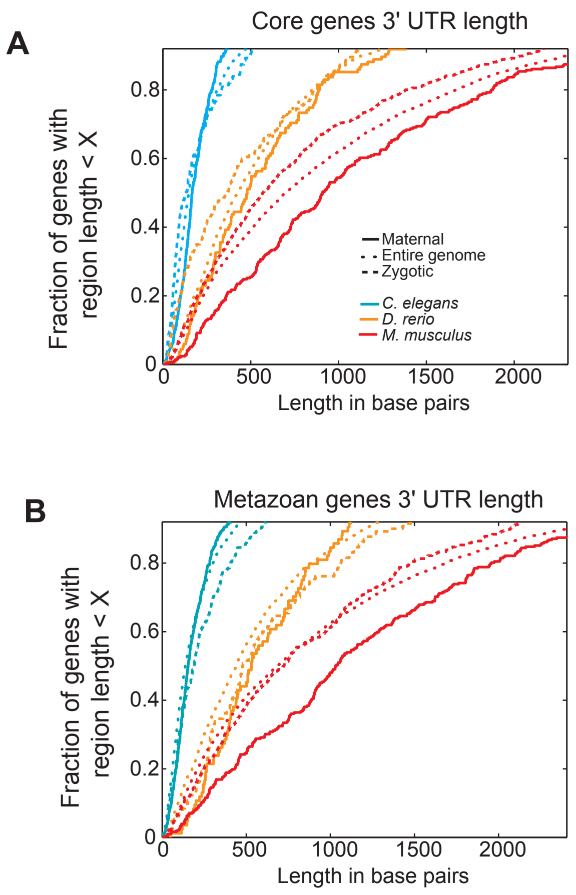
A
Core genes 3' UTR length
Fraction of genes with region length < X
0
0.2
0.4
0.6
0.8
0
500
1000
1500
2000
Length in base pairs
Maternal
Entire genome
Zygotic
C. elegans
D. rerio
M. musculus
B
Metazoan genes 3' UTR length
Fraction of genes with region length < X
0
0.2
0.4
0.6
0.8
0
500
1000
1500
2000
Length in base pairs

Figure S2.

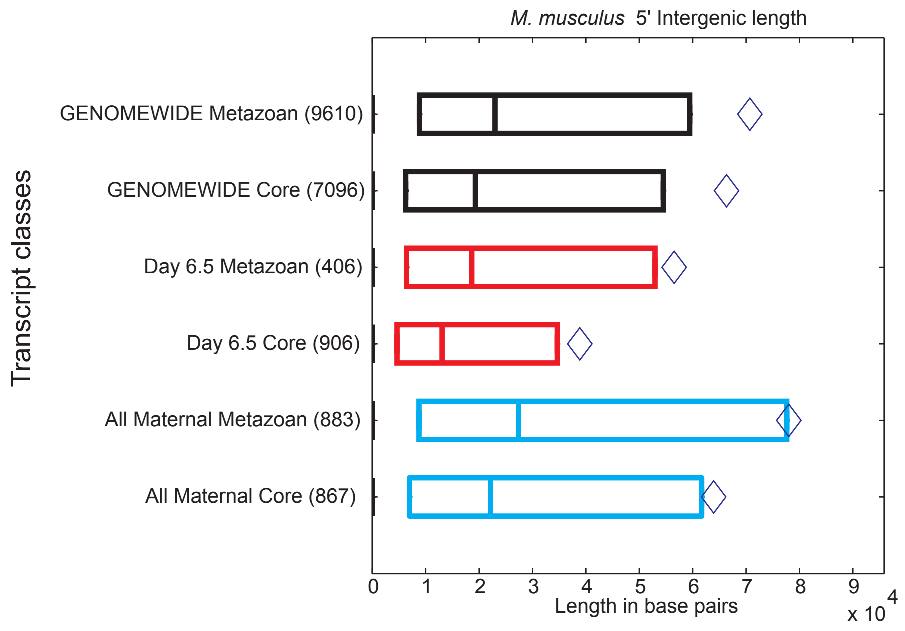
M. musculus 5' Intergenic length
GENOMEWIDE Metazoan (9610)
GENOMEWIDE Core (7096)
Day 6.5 Metazoan (406)
Day 6.5 Core (906)
All Maternal Metazoan (883)
All Maternal Core (867)
Transcript classes
0
1
2
3
4
5
6
7
8
9
Length in base pairs
x 10
4

Figure S3.

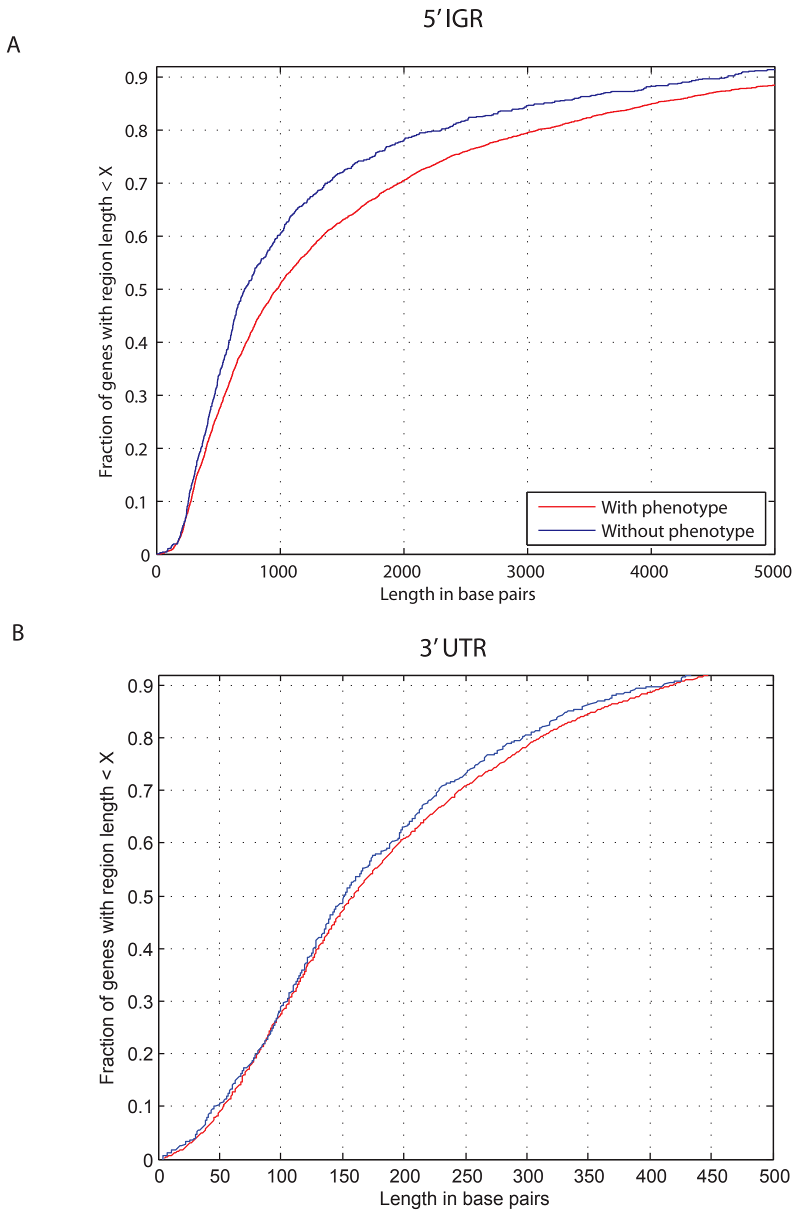
A
5′ IGR
Fraction of genes with region length < X
Length in base pairs
With phenotype
Without phenotype
0
0.1
0.2
0.3
0.4
0.5
0.6
0.7
0.8
0.9
0
1000
2000
3000
4000
5000
B
3′ UTR
Fraction of genes with region length < X
Length in base pairs
0
50
100
150
200
250
300
350
400
450
500

Figure S4.

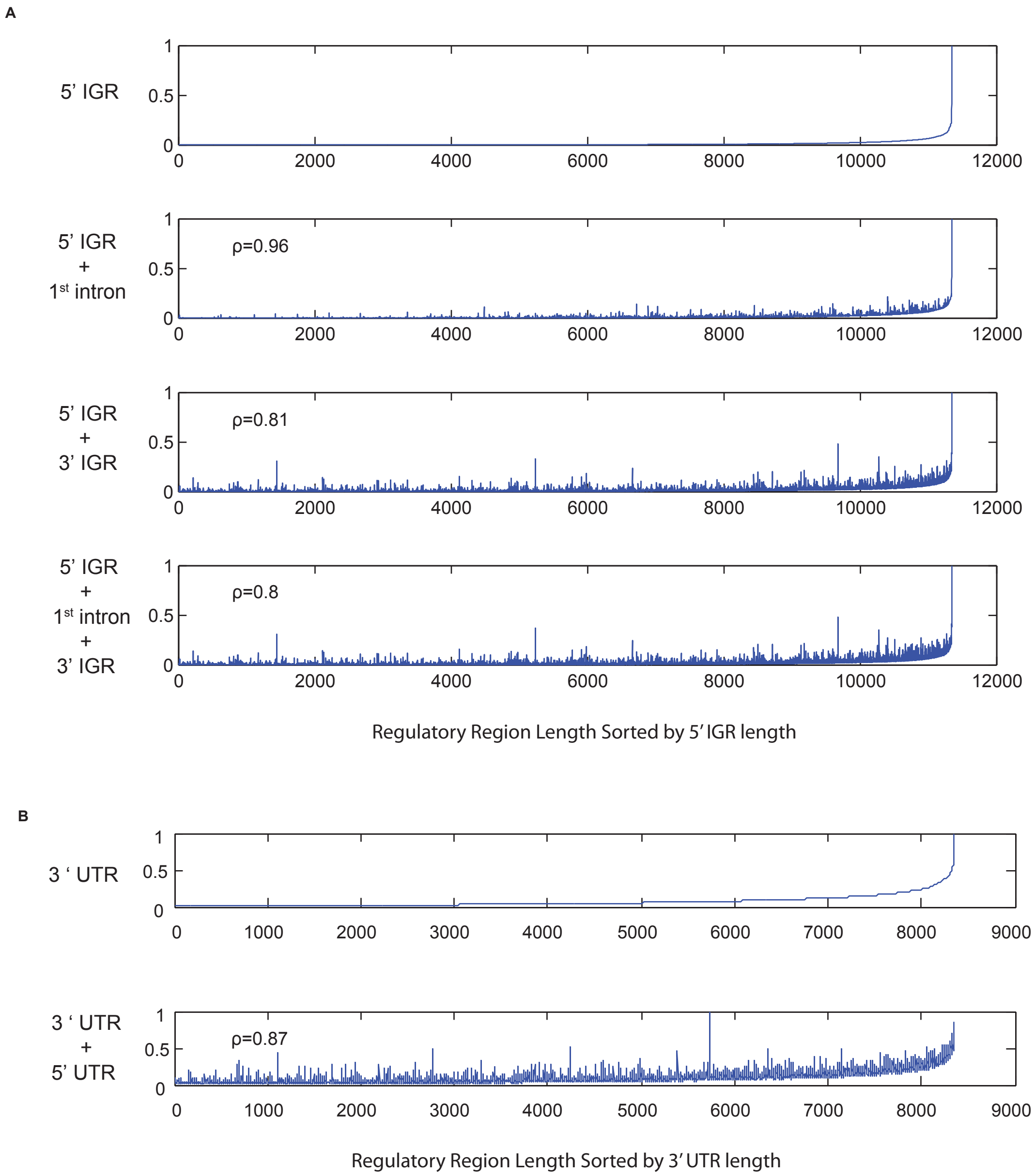
A
5' IGR
5' IGR + 1st intron
ρ=0.96
5' IGR + 3' IGR
ρ=0.81
5' IGR + 1st intron + 3' IGR
ρ=0.8
0
0.5
1
0
2000
4000
6000
8000
10000
12000
Regulatory Region Length Sorted by 5' IGR length
B
3 ' UTR
3 ' UTR + 5' UTR
ρ=0.87
0
1000
2000
3000
4000
5000
6000
7000
8000
9000
Regulatory Region Length Sorted by 3' UTR length

Figure S5.
